# Emergence of Colistin and Carbapenem Resistance in Extended-Spectrum β-Lactamase Producing *Klebsiella pneumoniae* Isolated from Chickens and Humans in Egypt

**DOI:** 10.3390/biology10050373

**Published:** 2021-04-26

**Authors:** Walid Elmonir, Norhan K. Abd El-Aziz, Yasmine H. Tartor, Samar M. Moustafa, Etab M. Abo Remela, Radwa Eissa, Hosam A. Saad, Ahmed Abdel Tawab

**Affiliations:** 1 Department of Hygiene and Preventive Medicine (Zoonoses), Faculty of Veterinary Medicine, Kafrelsheikh University, Kafrelsheikh 33516, Egypt; 2 Department of Microbiology, Faculty of Veterinary Medicine, Zagazig University, Zagazig 44511, Egypt; 3 Department of Zoonoses, Faculty of Veterinary Medicine, Benha University, Benha 13518, Egypt; samar.mmoustafa@yahoo.com; 4 Department of Bacteriology, Mycology and Immunology, Faculty of Veterinary Medicine, Kafrelsheikh University, Kafrelsheikh 33516, Egypt; etab_mh@yahoo.com; 5 Department of Biology, College of Science, Taibah University, Madina 42353, Saudi Arabia; 6 Department of Microbiology and Immunology, Faculty of Medicine, Tanta University, Tanta 31527, Egypt; radwa.eissa80@yahoo.com; 7 Department of Chemistry, College of Science, Taif University, P.O. Box 11099, Taif 21944, Saudi Arabia; h.saad@tu.edu.sa; 8 Department of Microbiology, Faculty of Medicine, Al Azhar University, Cairo 11884, Egypt; tawwab.206@azhar.edu.eg

**Keywords:** *K. pneumoniae*, colistin, ESBL, carbapenems, chickens, humans, environment

## Abstract

**Simple Summary:**

Carbapenems and colistin are reserved as the last-resort treatments of multidrug-resistant (MDR) infections in humans. Consequently, the emergence of carbapenem and colistin-resistant *Klebsiella pneumoniae* (*K. pneumoniae*) in poultry, contact workers and hospitalized patients is of grave concern for therapeutic options, and no data are available supporting this assumption on a regional or countrywide scale. We investigated the frequency and typing of extended-spectrum β-lactamase (ESBL) and carbapenemase-producing *K. pneumoniae* (ESBLK and CPK) in hospitalized patients, chickens from 10 poultry farms and their environment (water, food and litter) and farm workers in Egypt. All isolates from patients (13/90, 14.4%), workers (5/22, 22.7%), chickens (9/100, 9%) and the environment (10/60, 16.7%) harbored a single or multiple β-lactamase genes, *bla*_SHV_, *bla*_TEM_, *bla*_CTX-M1_ and *bla*_OXA-1_, often in combination with carbapenemase genes (*bla*_VIM_, *bla*_NDM-1_ or *bla*_IMP_; 45.9%), the *mcr*-1 gene (18.9%) or both (13.5%). Enterobacterial repetitive intergenic consensus (ERIC)-PCR genotyping highlighted potential inter and intraspecies clonal dissemination in the study area. The increased frequency and genetic relatedness of ESBLK and CPK from chickens and humans pose a public health threat that urges more prudent use of antimicrobials in chicken farms to avoid the propagation and expansion of both ESBLK and CPK from the chicken sources to humans.

**Abstract:**

This study investigated the frequency of carbapenem and colistin resistance in ESBL-producing *K. pneumoniae* (ESBLK) isolates recovered from chickens and their environment, contact farm workers and hospitalized patients in Egypt. Further, the phenotypic and genotypic relationships between the community and hospital-acquired *K. pneumoniae* isolates in the same geographical area were investigated. From 272 total samples, 37 (13.6%) *K. pneumoniae* isolates were identified, of which 20 (54.1%) were hypervirulent. All isolates (100%) were multidrug-resistant (MDR) with multiple antibiotic resistance (MAR) indices ranging from 0.19 to 0.94. Colistin-resistant isolates (18.9%) displayed colistin MIC values >2 μg/mL, all harbored the *mcr*-1 gene. All isolates from patients (13/90, 14.4%), workers (5/22, 22.7%), chickens (9/100, 9%) and the environment (10/60, 16.7%) harbored a single or multiple β-lactamase genes, *bla*_SHV_, *bla*_TEM_, *bla*_CTX-M1_ and *bla*_OXA-1_, often in combination with carbapenemase genes (*bla*_VIM_, *bla*_NDM-1_ or *bla*_IMP_; 45.9%), the *mcr*-1 gene (18.9%) or both (13.5%). Enterobacterial repetitive intergenic consensus (ERIC)–PCR genotyping revealed 24 distinct ERIC types (ETs) with a discrimination index of 0.961. Six ETs showed clusters of identical isolates from chicken and human sources. The increased frequency and genetic relatedness of ESBLK and carbapenemase-producing *K. pneumoniae* (CPK) from chickens and humans pose a public health threat that urge more prudent use of antimicrobials in chicken farms to avoid the propagation and expansion of both ESBLK and CPK from the chicken sources to humans.

## 1. Introduction

*Klebsiella pneumoniae* (*K. pneumoniae*) is a pervasive nosocomial and community associated-pathogen causing a wide range of infections in humans and animals [1,2]. Bacterial invasion in livestock animals poses a potential hazard to public health, as infected animals act as a reservoir of multidrug-resistant (MDR) isolates [1,3]. In Egypt, no regulations are controlling the use of antimicrobials in animals [4], which may be used as growth promoters or for the prevention and treatment of zoonotic diseases. This supports the evidence linking consumption of antimicrobials in livestock animals to their resistance in humans [5].

The emergence of extended-spectrum β-lactamase-producing *K. pneumoniae* (ESBLK) in Egypt is of serious concern. Previous studies suggested that food-producing animals, particularly chickens, have been considered a possible source for transmission of ESBLK to humans [6,7]. However, integrated studies on chickens and their environment, contact workers and hospitalized patients in the same area are still missing.

Carbapenems and polymyxin E (colistin) are considered the last-resort for treating infections caused by MDR *K. pneumoniae* isolates, particularly those producing extended-spectrum β-lactamase (ESBL) enzymes [8,9]. Carbapenem-resistant *K. pneumoniae* exhibited coresistance to a range of critically relevant antimicrobial classes resulting in few therapeutic possibilities [10]. The emergence of carbapenemases together with the *mcr-*1 colistin resistance gene constitutes a global risk to public health [11,12]. There are three mechanisms by which *K. pneumoniae* employs carbapenem resistance: (i) enzymatic hydrolysis via carbapenemases enzymes, (ii) overexpression of the efflux pump system and (iii) loss of porin expression [13]. Carbapenemases (i.e., Ambler molecular classes A, B and D β-lactamases) represent the most prevalent mechanism of carbapenem resistance. They hydrolyze a wide variety of β-lactams including penicillins, cephalosporins, monobactams, carbapenems and β-lactamases inhibitors through carbapenemase encoding genes, mainly of class B metallo-β-lactamases (MBL), including imipenem metallo-β-lactamases (*bla_IMP_*), New Delhi metallo-β-lactamases (*bla_NDM_*) and Verona integron-encoded metallo-β-lactamases (*bla_VIM_*) [14]. Carbapenemases are mostly detected in *K. pneumoniae*, with a lower extent in other enterobacterial species [15]. Although carbapenem and colistin-resistant *K. pneumoniae* isolates have been documented in numerous human studies in Egypt [8,16,17,18], no data are available on this issue from a veterinary overview. Moreover, the role of poultry and their environment in the maintenance and transmission of carbapenem-and colistin coresistant isolates is poorly explored.

Enterobacterial repetitive intergenic consensus polymerase chain reaction (ERIC-PCR) has been documented to have a virtuous differentiation power for molecular characterization and genotyping of bacterial isolates from human and animal origins [19,20]. Therefore, this study aimed to determine the frequency of carbapenem and colistin resistance in ESBLK isolates recovered from chickens and their environment, contact farm workers and hospitalized patients in Kafrelsheikh Governorate, Egypt, and to investigate the phenotypic and genotypic relationships between the community and hospital-acquired *K. pneumoniae* isolates in the same geographical area.

## 2. Materials and Methods

### 2.1. Study Design and Sampling

For determination of the potential risk for chicken-human cross-transmission, we detected the occurrence of carbapenemase and colistin resistance in ESBLK in hospitalized patients, workers who were in close contact with broiler chickens, broilers and their environment. The protocol of this study was approved by the ethics committee of Kafrelsheikh University, Kafrelsheikh, Egypt (KFS-2019/3). Written informed consent was attained from the patients, poultry workers and owners of farms for the participation in this study and the publication of any potentially identifiable data.

The sampling was carried out during the period from April 2019 to November 2019. Sputum and urine samples were collected from 90 hospitalized patients showing manifestations of respiratory and urinary tract infections. Moreover, 22 stool samples were collected from contact workers from 10 different broiler farms in Kafrelsheikh Governorate, Egypt. Additionally, 100 lung and trachea samples were collected from broilers showing respiratory manifestations. Furthermore, 60 environmental samples were included: two pooled samples for water, food and litter (*n* = 2 each) per farm were separately screened for carbapenem and colistin resistance in ESBLK.

### 2.2. Isolation and Identification of K. pneumoniae

Samples were directly streaked onto HiCrome™ Klebsiella selective agar (Himedia, India) at 37 °C for 24 h. The presumptive purple colonies were confirmed by growth on MacConkey’s agar and eosin methylene blue (Oxoid, Cambridge, UK) agar media and identified by biochemical tests [21]. The hypermucoviscous phenotype of colonies on an agar plate has been defined by a positive string test [22]. The 16S-23S rDNA internal transcribed spacer (ITS) of *K. pneumoniae* isolates were amplified with the species-specific primers reported previously [23]. *K. Pneumoniae* isolates grown on Columbia sheep blood agar (Oxoid, Cambridge, UK) plates for 24 h were subjected to matrix-assisted laser desorption ionization-time of flight mass spectrometry (MALDI-TOF MS) (BioMeri’eux, Marcy I’Etoile, France) identification using αcyano-4-hydroxycinnamic acid matrix solution as previously described [24]. MALDI-TOF MS running MYLA 3.1.0-15 software (BioM’erieux, Inc., Marcy I’Etoile, France) analyzed, compared the generated spectrum for each tested isolate with a library of standard reference spectra and calculated the confidence values.

### 2.3. Antimicrobial Susceptibility Testing

The disc diffusion test [25] was used for testing the susceptibility of *K. pneumoniae* isolates to a range of antimicrobials approved for both human and livestock use [26], including ampicillin (10 µg), amoxicillin-clavulanic acid (30 µg), ceftazidime (30 µg), cefepime (30 µg), amikacin (30 µg), nalidixic acid (30 µg), ciprofloxacin (5 µg), imipenem (10 µg), azithromycin (15 µg), aztreonam (30 µg), gentamicin (10 µg), tetracycline (30 µg), chloramphenicol (30 µg), nitrofurantoin (300 µg), trimethoprim-sulfamethoxazole (25 µg) and colistin (25 µg).

The broth microdilution method [27] was used for the determination of the minimum inhibitory concentrations (MICs) of colistin (Sigma-Aldrich, Seelze, Germany). A *K. pneumoniae* ATCC 700603 reference strain was included as quality control in the disc diffusion test and the fully colistin-susceptible *E. coli* ATCC 25922 and the *mcr*-1-positive *E. coli* NCTC 13846 with a colistin MIC of 4 mg/L were used in broth microdilution method.The results of antimicrobial susceptibility testing were interpreted according to the Clinical and Laboratory Standards Institute and European Committee on Antimicrobial Susceptibility Testing (EUCAST) joint subcommittee [28]. The multiple antibiotic resistance (MAR) index for each isolate was calculated according to Tambekaret al. [29].

### 2.4. Phenotypic Screening for ESBLs and Carbapenemases Production

ESBL production was screened using the CLSI ESBL confirmatory test (double disc synergy test) with cefotaxime (CTX, 30 µg) and ceftazidime (CAZ, 30 µg) antimicrobial discs alone, and in combination with clavulanic acid (CA, 10 µg) (Becton, Dickinson, Sparks, MD, USA). A positive ESBL production was considered when an increase in the inhibition zone diameter for CTX or the CAZ disc + CA was ≥5 mm of the diameter around the CTX or CAZ [30]. The modified Hodge test (MHT) was performed to screen for carbapenemase production according to CLSI guidelines [31]. The EDTA-imipenem synergy test was used for MBL carbapenemase identification [32]. *K. pneumoniae* ATCCBAA-1705 (carbapenemase-positive) and ATCC700603 (SHV-18 producer) reference strains were used as positive controls. A β-lactamase-negative *E. coli* ATCC25922 strain was used as a negative control. The ESBL- and carbapenemases-producing isolates were subjected to further analysis including detection of the resistance determinants, *mcr*-1gene in colistin-resistant isolates and ERIC-genotyping.

### 2.5. Detection of ESBL, Carbapenemase-Encoding Genes, and Colistin Resistance Determinant mcr-1

Genomic DNA was extracted from overnight cultures of all presumptive ESBLK and carbapenemase-producing *K. pneumoniae* (CPK) isolates using QIAamp DNA Mini kit (Qiagen GmbH, Hilden, Germany) according to the manufacturer’s instructions. The purity and concentrations of extracted DNA were assessed by using a nanodrop™ 2000 and 2000c (Thermo Fisher Scientific, Waltham, MA, USA). The ESBL genes (*bla*_SHV_, *bla*_CTX-M_, *bla*_TEM_ and *bla*_OXA-1_) were detected using the previously described multiplex PCR [33]. PCR amplification of carbapenemase genes (*bla_IMP_*, *bla_VIM_* and *bla_NDM1_*) was performed using specific primers [34,35,36] listed in Table S1. The uniplex PCR was carried out in a 25-µL reaction mixture containing 12.5 µL of EmeraldAmp Max PCR Master Mix (Takara, Shigino-higashi, Joto-ku, Osaka, Japan), 1 µL of each primer (20 pmol; Metabion GmbH, Germany), 5.5 µL of nuclease-free water and 5 µL of DNA template using the Applied Biosystems^®^ 2720 thermal cycler (Thermo Scientific, Waltham, MA, USA). The PCR products were electrophoresed on 1.5% agarose gel (Applichem GmbH, Darmstadt, Germany) containing 0.5 µg/mL ethidium bromide. The gel was photographed by Alpha Innotech gel documentation system (Biometra GmbH, Göttingen, Germany) and the data were analyzed through computer software.

Furthermore, colistin-resistant *K. pneumoniae* isolates were tested for the presence of the *mcr*-1 gene using the specific primers (Table S1) as described previously [37]. Genomic DNA from *mcr*-1-positive *E. coli* NCTC 13846 and colistin-susceptible *E. coli* ATCC 25922 were included in each run.

### 2.6. ERIC Genotyping

For analysis of fingerprinting profiles of different ESBL and carbapenemase-positive isolates, ERIC-PCR was applied using the ERIC-1R and ERIC-2 primers (Table S1), as previously described [38]. The ERIC-PCR band patterns were analyzed by GelJ software v.2.0 [39]. The comparison between fingerprinting profiles was conducted using the Dice coefficient, and a dendrogram was constructed using the unweighted pair group method with arithmetic mean. Simpson’s discrimination index for ERIC genotyping was estimated as previously described [40].

### 2.7. Data Analysis

Differences between frequencies of *K. pneumonia* in chicken and human samples were assessed by the chi-squared test. A univariate logistic regression model was used to estimate the odds ratios and significant associations between phenotypic and genetic antimicrobial resistance profiles and source (humans versus chickens) of the *K. pneumoniae* isolates. The analysis was done using SPSS v19 (IBM, Armonk, NY, USA), and associations at a *p*-value ≤ 0.05 were considered significant. To determine the distribution of the isolates from various hosts based on their antimicrobial resistance gene profiles, the occurrence of a particular gene in the isolates was entered as binary data (0 = absent, 1 = present), and this was used as inputs into non-metric multidimensional scaling (nMDS) analyses using the Sorensen distance. The nMDS biplot was produced to determine the association of the genes and isolates.

## 3. Results

### 3.1. Prevalence of K. pneumoniae in Chickens, Their Environment, Contact Workers and Hospitalized Patients in the Study Area

In total, 37 *K. pneumoniae* isolates were identified using the standard bacteriological tests, PCR amplification of 16S-23S rDNA ITS region (fragment size 130 bp) and MALDI-TOF MS. The latter provided correct species-level identification for *K. pneumoniae* isolates with a confidence value of 99.9%. As presented in Table 1, *K. pneumoniae* isolates were detected in 19 samples from diseased chickens (9/100; 9%) and their compartments (10/60; 16.7%), and 18 human samples including contact workers (5/22; 22.7%) and hospitalized patients (13/90; 14.4%). Six of ten poultry farms (60%) were positive for *K. pneumoniae* (Table S2). Of note, 20 isolates (54.1%) were hypervirulent, demonstrating a hypermucoviscous phenotype as confirmed by the string test (Table 2).

### 3.2. Antimicrobial Resistance of K. pneumoniae Isolates

All *K. pneumoniae* isolates were MDR, being resistant to 3–15 of the 16 tested antimicrobials with MAR indices ranged from 0.19 to 0. 94. The MDR isolates were assigned to a total of 32 distinct resistance patterns (Table 2). All hospitalized patients´ isolates showed high levels of MDR being resistant to 10–15 antimicrobials (MAR indices ranged from 0.63–0.94) and the farm workers’ isolates were resistant to 10–14 antimicrobials (MAR indices ranged from 0.63–0.88). The majority of chicken isolates (66.7%) were resistant to 11–13 antimicrobials with MAR indices 0.68–0.81. Moreover, 60% of the isolates from environment samples were resistant to 10–13 antimicrobials (MAR indices = 0.63–0.81). Overall, the isolates exhibited full resistance to ampicillin (100%) and 97.3% resistance to amoxicillin-clavulanic acid. Moreover, 94.6% of the isolates exhibited resistance to each of nalidixic acid and trimethoprim-sulfamethoxazole, whilst 86.5% were resistant to ceftazidime and cefepime. The resistance to tetracycline was found among 83.8% of the isolates, followed by nitrofurantoin (81.1%), ciprofloxacin (72.9%), chloramphenicol (70.3%), azithromycin (64.9%), amikacin (59.5%) and imipenem (45.9%). The lowest resistance rate was found to colistin (18.9%) followed by aztreonam (37.8%) and gentamicin (43.2%).

A univariate logistic regression model analysis (Table S3) showed that the acquisition of carbapenemase encoding genes (*bla*_VIM_ and *bla*_NDM_) were more likely to be associated with human isolates than chicken ones (*OR* = 10.6–36, *p* = 0.002–0.01). A similar association was recorded for imipenem phenotypic resistance (77.8% vs. 15.8%, *OR* = 18.7, *p* = 0.001). Additionally, human isolates were more likely resistant to azithromycin, aztreonam and gentamicin antimicrobials than chicken isolates (*OR* = 4.4–29.1, *p* = 0.003–0.04) (Table S3). There was no significant association with regard to other antimicrobial resistance genes or tested antimicrobials among detected isolates.

### 3.3. Distributions of ESBLs, Carbapenemase Genes and Colistin Resistance Determinant mcr-1 among K. pneumoniae Isolates

**Figure 1:**
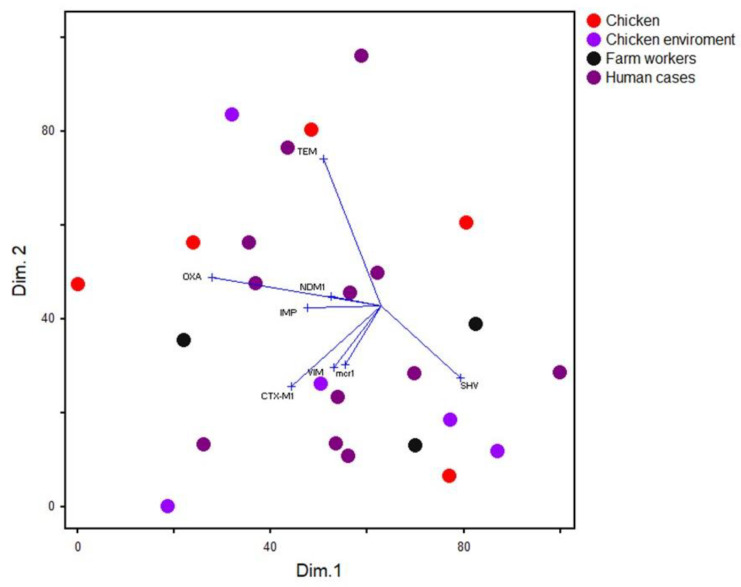
Non-metric multidimensional scaling biplot showing the overall distribution of isolates from various hosts based on the frequency distribution of antimicrobial resistance genes. Each dot refers to one isolate and the arrows refer to the association of each gene with either dimension 1 or 2.

Molecular testing revealed that among the 37 phenotypically positive isolates for ESBLs, 17 *bla*_SHV_, 14 *bla*_TEM_, 9 *bla*_CTX-M1_ and 6 *bla*_OXA-1_ producers were identified (Table 2). Seventeen isolates with decreased susceptibility to imipenem, and showed positive MHT, produced MBL carbapenemases and possessed *bla*_VIM_ (*n* = 13), *bla*_NDM-1_ (*n* = 12) and *bla*_IMP_ (*n* = 5). As depicted in Table 1, Table 2 and Table 3 and Figure 1, all CPK isolates harbored carbapenemase genes in combination with other ESBL genes. As presented in Table 3, 25 profiles were found for the distributions of ESBL and carbapenemase genes among ESBLK and CPK isolates. Importantly, five human isolates (13.5%) harbored ESBLs, carbapenemases genes and colistin resistance determinant *mcr-1*. Moreover, two isolates with *bla*_TEM_, *bla*_CTX_, *bla*_OXA-1_ and *bla*_SHV_ genotype profiles from chicken and their environmental samples also harbored the *mcr-*1 gene (Table 1, Table 2 and Table 3). Figure 1 presents the ESBL and carbapenemases genes found in humans, broilers and environment samples. In the broiler farms, the predominant ESBL and carbapenemases of ESBLK and CPK isolates from chickens and their environment were *bla*_SHV_ (11/19; 57.9%) and *bla*_NDM_ (2/19, 10.5%); *bla_S_*_HV_ (4/5, 80%), *bla*_VIM_ and *bla*_NDM_ (3/5; 60% each) were predominant in poultry workers. *bla*_SHV_, *bla*_TEM_ (7/13; 53.8% each) and *bla*_VIM_ (9/13; 69.2%) were the most frequent genes in hospitalized patients in the study area. As presented in Figure 1, the nMDS analysis of *K. pneumoniae* isolates (*n* = 37) displayed a non-specific clustering pattern, as the chicken and human isolates largely overlapped. However, 12 isolates among all analyzed origins were omitted from the analysis as they were identical and demonstrated unique patterns.

In the univariate analysis, *bla*_VIM_ and *bla*_NDM_ were significantly associated with human isolates with increased odds (*p* = 0.002, 0.01; *OR* = 36, 10.6; and 95% *CI* = 3.8–337.9, 1.9–60.2, respectively).

### 3.4. Genotyping and Epidemiological Association of Chicken and Human Isolates

**Figure 2:**
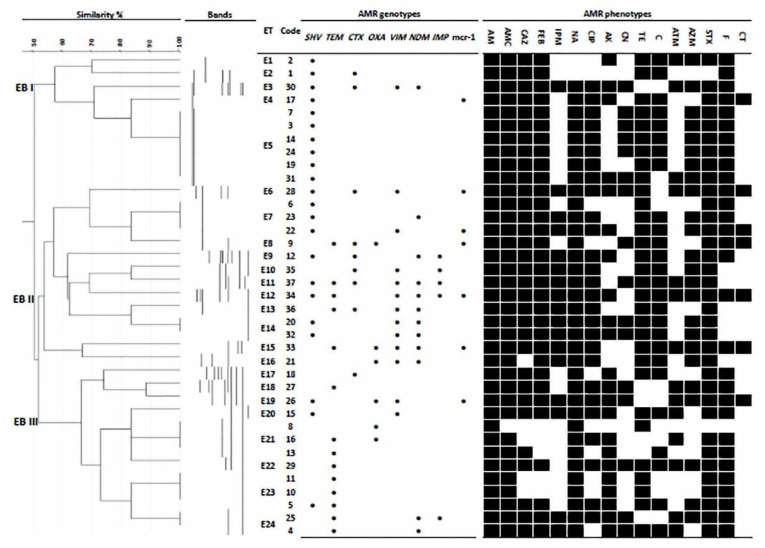
ERIC typing dendrogram of *K. pneumoniae* isolates from humans and chickens in the study area and their associated genetic and phenotypic antimicrobial resistance patterns. EB: ERIC branch; ET: ERIC type; black dot: positive for antibiotic resistance gene; black square: positive for antibiotic resistance phenotype; AMR: antimicrobial resistance; AMP: ampicillin; AMC: amoxicillin-clavulanic acid; CAZ: ceftazidime; FEB: cefepime; IPM: imipenem; NA: nalidixic acid; CIP: ciprofloxacin; AK: amikacin; CN: gentamicin; TE: tetracycline; C: chloramphenicol; ATM: aztreonam; AZM: azithromycin; SXT: trimethoprim-sulfamethoxazole; F: nitrofurantoin; CT: colistin.

The genetic relatedness and homology of ESBLK and CPK isolates from chickens, their environment, contact workers and hospitalized patients in the study area were investigated using ERIC-PCR. As revealed in Figure 2, ERIC genotyping classified 37 *K. Pneumoniae* isolates into three ERIC branches (EB): EBI, EBII and EBIII containing 13, 14 and 10 isolates of chickens and humans, respectively. These EBs contained 24 distinct ERIC types (ET) with band sizes ranged from 100 to 1400 bp with a discrimination index of 0.961. Six ETs (E5, E7, E14, E21, E23 and E24) showed clusters of two to six identical isolates per each ET (Figure 2). Of these, E5 contained four isolates (two from chickens, one from the chicken environment and one from farm workers) that shared identical antimicrobial resistance pattern (P16) (Figure 2).

## 4. Discussion

The emergence of colistin resistance in *K. pneumoniae* is a global public health concern, since this antibiotic is the last defense line against carbapenem-resistant isolates [41]. So far, there are no published data on colistin and carbapenem resistance in ESBLK isolated from chickens and their environment, community settings and hospitals in the same geographical region. In this study, *K. pneumoniae* was isolated from 9% of diseased chickens and 16.7% of environment samples, which is inconsistent with the findings reported in a previous study in Egypt, where *K. pneumoniae* was recovered from 35% of broiler chickens and 25% of water samples [42]. However, the prevalence of *K. pneumoniae* among contact poultry workers (22.7%) and hospitalized patients (14.4%) in the same region was lower than lately published reports in Egypt [7,42,43] or abroad, both in China [44] and Taiwan [45]. Hence, the direct transmission may be facilitated via close contact between chickens and humans as evidenced previously by Davis et al. [46]. The prevalence differences may be attributed to the geographical location, climatic circumstances, environmental contamination, sample types, chicken breed, management systems and growth conditions.

Antimicrobial resistance is an emergent affair of concern in human and veterinary medicine. In this study, *K. pneumoniae* resistance was detected most commonly to ampicillin (100%), followed by amoxicillin-clavulanic acid (94.6%), nalidixic acid and trimethoprim-sulfamethoxazole (94.6% each) and ceftazidime and cefepime (86.5% each). Almost half of the isolates (45.9%) showed imipenem resistance, whereas the lowest resistance rates were observed against colistin (18.9%), aztreonam (37.8%) and gentamicin (43.2%). In China, a previous study reported higher resistance rates of *K. pneumoniae* human isolates to β-lactams, quinolones and carbapenems, while all isolates exhibited 100% susceptibility to colistin [47]. Even worse, an earlier study in Shandong province, China documented pan drug-resistant *K. pneumoniae* isolates from both patients and chickens [48]. This disparity in resistance may be related to the antimicrobial agent frequently used for treatment in diverse geographical areas.

*K. pneumoniae* is one of the foremost imperative causes of MDR infections all over the world, resulting in inflated healthcare expenses and high mortalities [49]. Here, all *K. pneumoniae* isolates were MDR, being resistant to 3–15 commonly used antimicrobial agents either in Egypt or worldwide of diverse antimicrobial classes. Similarly in Indonesia, all *K. pneumoniae* isolates from chicken farms showed an MDR pattern [50], whereas the proportion of MDR *K. Pneumoniae* in Indonesian hospitalized patients was 54.49% [51]. However, 96% of *K. pneumoniae* isolates from humans and livestock including poultry demonstrated a nonwild-type phenotype to ≥ three antimicrobial classes in rural Cambodia [52]. The MDR pattern may be accredited to the unregulated use of antimicrobials in human and animal medicine [53], the facility in purchasing antimicrobials freely without prescription and the vertical or horizontal transmission of antimicrobial resistance genes via plasmids from humans to animals and vice versa [54]. Thus, the high level of MDR *K. pneumoniae* isolates warrants a commitment to establish purposeful antimicrobial stewardship.

ESBL-producing *Enterobacteriaceae* are virtually resistant to all β-lactams and may compel the use of last-resort antimicrobials as carbapenems and colistin [55]. Initially, hospital-associated *K. pneumoniae* was reported as an ESBL producer and, thereafter, community-acquired infections have emerged worldwide [56,57]. Herein, irrespective of the source, all *K. pneumoniae* isolates (*n =* 37) were phenotypically confirmed as ESBLs. According to PCR results, *bla*_SHV_ was the slightly predominant β-lactamase gene (45.95%), followed by *bla*_TEM_ (37.84%), *bla*_CTX-M1_ (24.32%) and *bla*_OXA-1_ (16.22%). Generally, it was documented that most *K. pneumoniae* isolates harbored SH-based non-ESBL β-lactamases as *bla*_SHV_ [58]. In Germany, all *K. pneumoniae* isolates from broilers and their environment carried the same ESBL-gene, *bla*_SHV−2_ [59]. In contrast, in Indonesia, *bla*_TEM_ (100%) was detected in all *K. pneumoniae* isolates of chicken origin, followed by *bla*_CTX-M_ (90.9%). However, only 9.1% of the isolates harbored *bla*_SHV_ [50].

In the current study, 17 out of 37 *K. pneumoniae* isolates were imipenem-resistant as well as MHT-positive (45.95%). Our CPK isolates possessed MBL carbapenemases comprising *bla*_VIM_ (76.47%), *bla*_NDM-1_(70.59%) and *bla*_IMP_ (29.41%). Concerning the source, the dominant enzymatic mechanism of carbapenemase resistance was *bla*_NDM_ (10.5%) in broiler farms, whereas *bla*_NDM_ and *bla*_VIM_ (60% each) were the most frequent in poultry workers. Moreover, *bla*_VIM_ (69.2%) was the predominant gene in the hospitalized patients in the study area. Thus, *bla*_VIM_ was frequently involved in causing carbapenem resistance in humans. Likewise, CPK occurred at a relatively high rate amongst broilers (42.86%), drinking water (60%) and contact workers (56%) at poultry farms in Egypt, all of them possessed *bla*_NDM_ [42]. However, an earlier report in Iran [60] was discordant with our results, in which *bla*_NDM-1_ was mostly detected in *K. pneumoniae* isolates from humans (75%), while *bla*_VIM_ was not found in any examined CPK isolates.

Globally, in hospitalized patients, an elevated rate of carbapenem resistance was reported in Greece (68%; NDM, OXA-48 and KPC), followed by India (54%; NDM, OXA-48 and KPC) and eastern Mediterranean regions (54%; NDM and OXA-48) [61], the USA and China (11% each; NDM, OXA−48 and KPC) and Africa (4%; NDM and OXA-48) [62].

In this study, cocarriage of ESBLs and carbapenem resistance genes in *K. pneumoniae* isolates was 45.9%, ESBLs and colistin resistance genes was 18.9% and carbapenem and colistin resistance genes was 13.5%. As reported previously, 90% of CPK isolates had the carbapenemase phenotype, of which 50% were ESBL-producers [63]. In France, the coxistence of ESBL genes and the *mcr-*1 colistin resistance gene was reported in a hospitalized patient [64]. Also, a recent study in Iran encountered chromosomal-mediated colistin resistance among CPK isolates in hospitalized patients with bloodstream infections [65]. Consistent with our results, a recent study in the United States documented the coexistence of carbapenem and colistin resistance in 13% of *K. pneumoniae* isolates from hospitalized patients [66]. The most striking finding in this study was the cocarriage of ESBLs, carbapenem and colistin resistance genes in 13.5% of *K. pneumoniae* isolates from poultry workers (*n* = 1/22; 4.5%) and hospitalized patients (*n* = 4/90; 4.4%), as the first report in Egypt. The extensive use of carbapenems results in selection pressure and simplifies the spread of carbapenem-resistant isolates, for which colistin is one of the last treatment choices. Further colistin resistance in CPK, such as the reported outbreak in hospitalized patients in Germany [67], leaves limited treatment options, among them fosfomycin and tigecycline. Thus, regimens of colistin combined with carbapenem display a high level of synergism even in the existence of colistin resistance [68].

In this study, we present the first report in the genetic relatedness of community and hospital-acquired *K. pneumoniae* isolates using ERIC-PCR. In this context, data fingerprints revealed pronounced similarities between *K. pneumoniae* isolates recovered from chicken farms and hospitalized patients. These may originate from a common ancestral strain, demonstrating cross-transmission between chickens and humans. A recent study in Iran showed immense genetic diversity among *K. pneumoniae* isolates from hospitalized patients [69]. Moreover, another Indian study demonstrated heterogeneous *K. pneumoniae* human isolates while using ERIC-PCR, suggesting the involvement of multiple subtypes in infection [70]. In light of our results, additional studies are needed to confirm the transmission between poultry and humans and to explain the direction and machinery of transmission.

The limitation of this study is due to restricted funding resources. Therefore, we investigated the genetic relatedness among the community and hospital-acquired ESBL producing *K. pneumoniae* isolates based on ERIC-PCR typing, which is a less expensive and less laborious approach in routine diagnosis that allows rapid genotyping with a high discriminatory potential, and thus is more suitable on a local scale in developing countries. However, using advanced techniques such as multilocus sequence typing (MLST) and pulsed-field gel electrophoresis (PFGE) would add more sensitivity and accuracy to the results and would allow comparison among different typing methods simultaneously. Future studies are warranted in this direction.

## 5. Conclusions

This is the first, at least in Egypt, of the coexistence of ESBLs, carbapenem and colistin resistance in *K. pneumoniae* isolates recovered from poultry workers and hospitalized patients. Moreover, our findings demonstrate great similarities between community and hospital-acquired *K. pneumoniae* isolates, which warrants the need to improve farm management strategies and implementation of proper hygiene practices in the study area.

## Figures and Tables

**Table 1:** Frequency distribution of 37 multidrug-resistant *K. pneumoniae* isolates recovered from broiler farms and human cases in the study area.

Isolates (%) *	No. of Isolates (%)						Chicken vs. Human	
	Chickens			Humans				
	C (*n* = 100)	Ce (*n* = 60)	TC (*n* = 160)	Cw (*n* = 22)	Hc (*n* = 90)	TH (*n* = 112)	X^2^	*p*-Value
MDR-*K. pneumoniae* (100)	9 (9)	10 (16.7)	19 (11.9)	5 (22.7)	13 (14.4)	18 (16.1)	0.9	0.3
ESBL-*K. pneumoniae* (100)	9 (9)	10 (16.7)	19 (11.9)	5 (22.7)	13 (14.4)	18 (16.1)	0.9	0.3
CR-*K. pneumoniae* (45.9)	1 (1)	2 (3.3)	3 (1.9)	4 (18.2)	10 (11.1)	14 (12.5)	12.7	>0.001
CTR-*K. pneumoniae* (18.9)	1 (1)	1 (1.7)	2 (1.25)	1 (4.5)	4 (4.4)	5 (4.5)	2.7	0.1
ESBL and CR-*K. pneumoniae* (45.9)	1 (1)	2 (3.3)	3 (1.9)	4 (18.2)	10 (11.1)	14 (12.5)	12.7	>0.001
ESBL and CTR-*K. pneumoniae* (18.9)	1(1)	1 (1.7)	2 (1.25)	1 (4.5)	4 (4.4)	5 (4.5)	2.7	0.1
CR and CTR-*K. pneumoniae* (13.5)	0	0	0	1 (4.5)	4 (4.4)	5 (4.5)	-	-
ESBL, CR and CTR-*K. pneumoniae* (13.5)	0	0	0	1 (4.5)	4 (4.4)	5 (4.5)	-	-

* Percentage was calculated from total isolates; C: diseased chicken; Ce: chicken environment samples; TC: total chicken samples; Cw: chicken worker; Hc: human case; TH: total human samples; MDR: multidrug-resistant; ESBL: extended-spectrum β-lactamases; CR: carbapenemases-producing; CTR: colistin resistant; X^2^: chi-square.

**Table 2:** Antimicrobial resistance phenotypes, their associated genes and ERIC fingerprinting patterns of hypervirulent ESBL and carbapenemase-producing *K. pneumoniae* isolates from chickens and humans in this study.

Pattern No.	Source	Code No.	Antimicrobial Resistance Pattern	MAR Index	Colistin MIC (mg/L)	Genotype	ERIC Fingerprint
							(Band Size)
1	Hc	**34**	Amp, AMC, CAZ, FEP, IPM, NA, CIP, AK, TE, C, ATM, AZM, SXT, F, CT	0.94	16	*bla*_SHV_, *bla*_TEM_, *bla*_VIM_, *bla*_NDM-1_, *bla*_IMP_, *mcr-*1	E12 (109, 312, 423, 878, 1000, 1225)
2	Cw	22	Amp, AMC, CAZ, FEP, IPM, NA, CIP, AK, TE, C, AZM, SXT, F, CT	0.88	8	*bla*_SHV_, *bla*_VIM_, *mcr-*1	E7 (872)
3	Cw	23	Amp, AMC, CAZ, FEP, NA, IPM, CIP, TE, C, AZM, SXT, F	0.75	1	*bla*_SHV_, *bla*_NDM-1_	E7 (872)
4	C	6	Amp, AMC, CAZ, FEP, NA, TE, SXT, F	0.5	0.25	*bla*_SHV_	E7 (878)
5	Hc	28	Amp, AMC, CAZ, FEP, CN, IPM, NA, CIP, AK, TE, ATM, AZM, SXT, F, CT	0.94	32	*bla*_SHV_, *bla*_CTX-M1_, *bla*_VIM_, *mcr-*1	E6 (298, 428, 860, 1148)
6	Hc	29	Amp, AMC, CAZ, FEP, CN, NA, CIP, AK, TE, C, ATM, AZM, SXT, F	0.88	0.5	*bla*_TEM_	E22 (157, 305, 401)
7	C	4	Amp, AMC, CAZ, FEP, CN, IPM, NA, AK, TE, C, ATM, SXT, F	0.81	0.25	*bla*_TEM_, *bla*_NDM-1_	E24 (159, 412)
8	Hc	25	Amp, AMC, CAZ, FEP, CN, IPM, NA, CIP, AK, ATM, AZM, SXT, F	0.81	1	*bla*_TEM_, *bla*_NDM-1_, *bla*_IMP_	E24 (150, 380)
9	Ce	12	Amp, AMC, CAZ, FEP, IPM, NA, CIP, AK, TE, C, AZM, SXT, F	0.81	0.5	*bla*_SHV_, *bla*_CTX-M1_, *bla*_NDM-1_, *bla*_IMP_	E9 (127, 199, 294, 352, 436, 489, 748)
10	Ce	15	Amp, AMC, CAZ, FEP, IPM, NA, CIP, AK, TE, C, ATM, SXT, F	0.81	0.25	*bla*_SHV_, *bla*_VIM_	E20 (116, 168, 312)
**11**	Hc	**30**	Amp, AMC, CAZ, FEP, CN, IPM, NA, CIP, AK, TE, C, AZM, SXT	0.81	2	*bla*_SHV_, *bla*_VIM_, *bla*_NDM-1_	E3 (144, 169, 320, 340, 432, 1205, 1346) E14 (107)
	Cw	**20**			1		
12	Hc	**32**	Amp, AMC, CAZ, FEP, CN, IPM, NA, CIP, AK, ATM, AZM, SXT, F	0.81	2	*bla*_SHV_, *bla*_CTX-M1_, *bla*_VIM_, *bla*_NDM-1_	E14 (117)
13	Hc	**26**	Amp, AMC, CAZ, FEP, CN, IPM, NA, CIP, AK, ATM, AZM, SXT, F, CT	0.88	16	*bla*_SHV_, *bla_OXA-1_*, *bla*_VIM_, *mcr-*1	E19 (159, 244, 307, 429, 680)
14	Hc	**31**	Amp, AMC, CAZ, FEP, CN, NA, CIP, AK, ATM, TE, AZM, SXT, F	0.81	1	*bla*_SHV_	E5 (1187, 1307)
15	Ce	**19**	Amp, AMC, CAZ, FEP, NA, TE, CIP, C, AZM, SXT, F	0.69	2	*bla*_SHV_	E5 (1205, 1353)
**16**	C	**7**	Amp, AMC, CAZ, FEP, CN, NA, CIP, TE, C, AZM, SXT, F	0.75	2	*bla*_SHV_	E5 (1196, 1320) E5 (1214, 1353) E5 (1200,1333) E5 (1214, 1353)
	C	**3**			2		
	Ce	**14**			1		
	Cw	**24**			2		
17	C	5	Amp, AMC, CAZ, FEP, NA, CIP, AK, TE, C, AZM, SXT, F	0.75	0.5	*bla*_SHV_, *bla*_TEM_	E23 (157)
**18**	Ce	10	Amp, AMC, NA, AK, TE, SXT, F	0.44	0.5	*bla*_TEM_	E23 (159)
	Ce	11			1		
19	Hc	**37**	Amp, AMC, CAZ, FEP, CN, IPM, NA, CIP, ATM, TE, C, AZM, SXT	0.81	1	*bla*_SHV_, *bla*_TEM_, *bla*_CTX-M1_, *bla*_VIM_, *bla*_NDM-1_, *bla*_IMP_	E11 (188, 306)
20	Ce	**17**	Amp, AMC, CAZ, FEP, NA, CIP, AK, TE, C, SXT, F, CT	0.75	4	*bla*_SHV_, *mcr-*1	E4 (1360)
21	Hc	**27**	Amp, AMC, CAZ, FEP, NA, AK, CN, CIP, ATM, AZM, SXT, F	0.75	2	*bla*_TEM_	E18 (160, 254, 301, 369, 424, 703, 884, 1197)
22	C	**2**	Amp, AMC, CAZ, FEP, TE, AK, C, ATM, AZM, SXT, F	0.69	1	*bla*_SHV_	E1 (679)
23	C	9	Amp, AMC, CAZ, FEP, NA, TE, C, CN, SXT, F, CT	0.69	8	*bla*_TEM_, *bla*_CTX-M1_, *bla_OXA-1_*, *mcr-*1	E8 (891)
24	Hc	**35**	Amp, AMC, CAZ, IPM, FEP, NA, TE, CIP, C, AZM, SXT	0.69	2	*bla*_CTX-M1_, *bla*_VIM_, *bla*_IMP_	E10 (107, 188, 377, 492)
25	Ce	**18**	Amp, AMC, CAZ, FEP, NA, TE, AK, C, SXT, F	0.63	1	*bla*_CTX-M1_	E17 (189, 266, 331, 373, 488, 565, 623, 964)
26	Hc	**33**	Amp, AMC, CAZ, FEP, NA, IPM, CIP, TE, C, ATM, AZM, SXT, F, CT	0.88	16	*bla*_TEM_, *bla_OXA-1_*, *bla*_VIM_, *bla*_NDM-1_, *mcr-*1	E15 (167, 211, 316)
27	Cw	21	Amp, AMC, FEP, NA, IPM, CIP, TE, AZM, C, SXT	0.63	0.25	*bla_OXA-1_*, *bla*_VIM_, *bla*_NDM-1_	E16 (396, 767, 1357)
28	Hc	**36**	Amp, AMC, CAZ, FEP, NA, IPM, CIP, TE, C, SXT	0.63	2	*bla*_TEM_, *bla*_CTX-M1_, *bla*_VIM_, *bla*_NDM-1_	E13 (107, 878)
29	Ce	13	Amp, AMC, CAZ, NA, AK, C, SXT, F	0.5	0.5	*bla*_TEM_	E21(170, 320, 459)
30	Ce	16	Amp, AMC, CIP, NA, AK, ATM, SXT, F	0.5	1	*bla*_TEM_, *bla*_OXA_	E21(169, 320, 462)
31	C	8	AMP, NA, TE	0.19	0.25	*bla*_OXA-1_	E21(170, 320, 466)
32	C	**1**	Amp, AMC, CAZ, FEB, TE, C, F	0.44	1	*bla*_SHV_, *bla*_CTX-M1_	E2 (297, 409, 700, 1158)

PT: Phenotypic resistance pattern; AMP: ampicillin; AMC: amoxicillin-clavulanic acid; CAZ: ceftazidime; FEB: cefepime; IPM: imipenem; NA: nalidixic acid; CIP: ciprofloxacin; AK: amikacin; CN: gentamicin; TE: tetracycline; C: chloramphenicol; ATM: aztreonam; AZM: azithromycin; SXT: trimethoprim-sulfamethoxazole; F: nitrofurantoin; CT: colistin; MAR: multiple antibiotic resistance index; Hc: human case; Cw: chicken worker; C: chicken; Ce: chicken environment. Patterns with bold numbers were found in more than one isolate. Isolates with bold code numbers are hypervirulent.

**Table 3:** Distribution of extended-spectrum β-lactamase, carbapenemase genes and colistin resistance determinant *mcr*-1 among 37 *K. pneumoniae* isolates from chickens and humans.

No.	Isolates Group and Genotypes	Total	Chickens			Humans		
			C	Ce	TC	Cw	Hc	TH
	**ESBL-producing isolates**							
1	*bla*_SHV_	8 (21.6)	4	2	6 (75)	1	1	2 (25)
2	*bla*_SHV_, *bla*_CTX-M1_	1 (2.7)	1		1 (100)			
3	*bla*_SHV_, *bla*_TEM_	1 (2.7)	1		1 (100)			
4	*bla*_TEM_	5 (13.5)		3	3 (60)		2	2 (40)
5	*bla*_TEM_, *bla*_OXA-1_	1 (2.7)		1	1 (100)			
6	*bla*_CTX-M1_	1 (2.7)		1	1 (100)			
7	*bla*_OXA-1_	1 (2.7)	1		1 (100)			
	**ESBL- and CR-producing isolates**							
8	*bla*_SHV_, *bla*_VIM_	1 (2.7)		1	1 (100)			
9	*bla*_SHV_, *bla*_NDM-1_	1 (2.7)				1		1 (100)
10	*bla*_SHV_, *bla*_VIM_, *bla*_NDM-1_	2 (5.4)				1	1	2 (100)
11	*bla*_SHV_, *bla*_CTX-M1_, *bla*_VIM_, *bla*_NDM-1_	1 (2.7)					1	1 (100)
12	*bla*_SHV_, *bla*_CTX-M1_, *bla*_NDM-1_, *bla*_IMP_	1 (2.7)		1	1 (100)			
13	*bla*_SHV_, *bla*_TEM_, *bla*_CTX-M1_, *bla*_VIM_, *bla*_NDM-1_, *bla*_IMP_	1 (2.7)					1	
14	*bla*_TEM_, *bla*_NDM-1_	1 (2.7)	1		1 (100)			
15	*bla*_TEM_, *bla*_NDM-1_, *bla*_IMP_	1 (2.7)					1	1 (100)
16	*bla*_TEM_, *bla*_CTX-M1_, *bla*_VIM_, *bla*_NDM-1_	1 (2.7)					1	1 (100)
17	*bla*_CTX-M1_, *bla*_VIM_, *bla*_IMP_	1 (2.7)					1	1 (100)
18	*bla*_OXA_, *bla*_VIM_, *bla*_NDM-1_	1 (2.7)				1		1 (100)
	**ESBL-producing and CTR isolates**							
19	*bla*_SHV_, *mcr*-1	1 (2.7)		1	1 (100)			
20	*bla*_TEM_, *bla*_CTX-M1_, *bla*_OXA-1_, *mcr*-1	1 (2.7)	1		1 (100)			
	**ESBL-, CR- producing and CTR isolates**							
21	*bla*_SHV_, *bla*_VIM_, *mcr*-1	1 (2.7)				1		1 (100)
22	*bla*_SHV_, *bla*_CTX-M1_, *bla*_VIM_, *mcr*-1	1 (2.7)					1	1 (100)
23	*bla*_SHV_, *bla*_OXA-1_, *bla*_VIM_, *mcr*-1	1 (2.7)					1	
24	*bla*_SHV_, *bla*_TEM_, *bla*_VIM_, *bla*_NDM-1_, *bla*_IMP_, *mcr*-1	1 (2.7)					1	
25	*bla*_TEM_, *bla*_OXA-1_, *bla*_VIM_, *bla*_NDM-1_, *mcr*-1	1 (2.7)					1	1 (100)

ESBL: extended-spectrum β-lactamase; CR: carbapenemases-producing; CTR: colistin resistant; C: chicken; Ce: chicken environment; TC: total chicken samples; Cw: chicken worker; Hc: human case; TH: total human samples.

## Supplementary Materials

The following are available online at www.mdpi.com/xxx/s1.Table S1: Oligonucleotide primer sequences used for PCR assays. Table S2: Frequency distribution of *K. pneumoniae* isolates recovered from broiler farms and human workers in the study area. Table S3: Source associated variations in phenotypic and genetic antibiotic resistance traits of *K. pneumoniae* isolates from chickens and humans in this study.
