# Supplementary material for: Emergence of Colistin and Carbapenem Resistance in Extended-Spectrum β-Lactamase Producing Klebsiella pneumoniae Isolated from Chickens and Humans in Egypt
Source: Biology (Basel). 2021 Apr 26;10(5):373. doi: 10.3390/biology10050373 (PMC8146310; doi:10.3390/biology10050373)
Supplement: Supplementary file 1 [file biology-10-00373-s001.zip › biology-1188930-supplementary.pdf]

biology MDPI

Table S1. Oligonucleotide primer sequences used for PCR assays.

Target Gene	Primer	Oligonucleotide Sequence (5′→3′)	Product Size (bp)§	Annealing Temperature (°C)	References
K. pneumoniae 16S-23S ITS*	*K. pneumoniae* Pf<br>*K. pneumoniae* Pr1	ATTTGAAGAGGTTGCAAACGAT<br>TTCACTCTGAAGTTTTCTTGTGTTC	130	57	[23]
bla$_{OXA-1}$	OXA-F<br>OXA-R	GGCACCAGATTCAACTTTCAAG<br>GACCCCAAGTTTCCTGTAAGTG	564	61	[33]
bla$_{CTX-M1}$	CTX-M-1-F<br>CTX-M-1-R	TTAGGAAGTGTGCCGCTGTA<br>CGGTTTTATCCCCCACAAC	655		
bla$_{SHV}$	SHV-F<br>SHV-R	AGCCGCTTGAGCAAATTAAAC<br>ATCCCGCAGATAAATCACCAC	713		
bla$_{TEM}$	TEM-F<br>TEM-R	CATTTCCGTGTCGCCCTTATTC<br>CGTTCATCCATAGTTGCCTGAC	800		
bla$_{IMP}$	IMP1-F<br>IMP1-R	CATGGTTTGGTGGTTCTTGT<br>ATAATTTGGCGGACTTTGGC	488	53	[34]
bla$_{VIM}$	VIM-F<br>VIM-R	AGTGGTGAGTATCCGACA<br>ATGAAAGTGCGTGGAGAC	280	52	[35]
bla$_{NDM1}$	NDM-1-F<br>NDM-1-R	GGCGGAATGGCTCATCACGA<br>CGCAACACAGCCTGACTTTC	287	55	[36]
mcr-1	CLR5-F<br>CLR5-R	CGGTCAGTCCGTTTGTTC<br>CTTGGTCGGTCTGTAGGG	309	55	[37]
ERIC consensus	ERIC1R<br>ERIC2	ATGTAAGCTCCTGGGGATTCAC<br>AAGTAAGTGACTGGGGTGAGCG	Variable	52	[38]

* ITS, Internal transcribed spacer; § bp, base pair.

Table S2. Frequency distribution of *K. pneumoniae* isolates recovered from broiler farms and human workers in the study area.

Farms	Chicken						Cw**
	C	Ce*				TC	
		Water	Food	Litter	Total		
1	2	1	0	1	2	4	1
2	0	0	0	0	0	0	0
3	3	2	1	1	4	7	2/3
4	1	1	0	0	1	2	1
5	1	0	0	1	1	2	0
6	1	0	0	1	1	2	1
7	0	0	0	0	0	0	0
8	0	0	0	0	0	0	0/3
9	0	0	0	0	0	0	0
10	1	0	0	1	1	2	0
Total (%)	9/100 (9)	4/20 (20)	1/20 (5)	5/20 (25)	10/60 (16.7)	19/160 (11.9)	5/22 (22.7)

C: diseased chicken; Ce: chicken environment samples; TC: total chicken samples.

* Two pooled (a pool of three) samples for each type per farm; ** Cw: stool samples of chicken workers in broilers farms

Table S3. Source associated variations in phenotypic and genetic antibiotic resistance traits of *K. pneumoniae* isolates from chickens and humans in this study.

Variables		Source	Positive	OR	*p-Value	95% CI
Carbapenemase encoding gene	bla_{VIM}	Chicken	1/19 (5.3)	-	-	-
		Human	12/18 (66.7)	36	0.002	3.8–337.9
	bla_{NDM}	Chicken	2/19 (10.5)	-	-	-
		Human	10/18 (55.6)	10.6	0.01	1.9–60.2
Phenotypic resistance	Imipenem	Chicken	3/19 (15.8)	-	-	-
		Human	14/18 (77.8)	18.7	0.001	3.6–98.2
	Gentamicin	Chicken	5/19 (26.3)	-	-	-
		Human	11/18 (61.1)	4.4	0.04	1.1–17.7
	Aztreonam	Chicken	4/19 (21.1)	-	-	-
		Human	10/18 (55.6)	4.7	0.04	1.1–19.8
	Azithromycin	Chicken	7/19 (36.8)	-	-	-
		Human	17/18 (94.4)	29.1	0.003	3.2–268.8

* There was no significant association with regard to other antibiotic resistance genes or tested antimicrobials among the detected isolates. OR: Odds ration; CI: Confidence interval. p-value is considered significant at ≤0.05.

References

23. Liu, Y.; Liu, C.; Zheng, W.; Zhang, X.; Yu, J.; Gao, Q.; Hou, Y.; Huang, X. PCR detection of *Klebsiella pneumoniae* in infant formula based on 16S-23S internal transcribed spacer. *Int. J. Food Microbiol.* **2008**, *125*, 230–235, doi:10.1016/j.ijfoodmicro.2008.03.005.

33. Ogutu, J.O.; Zhang, Q.; Huang, Y.; Yan, H.; Su, L.; Gao, B.; Zhang, W.; Zhao, J.; Cai, W.; Li, W.; et al. Development of a multiplex PCR system and its application in detection of bla_{SHV}, bla_{TEM}, $bla_{CTX\text{-}M\text{-}1}$, $bla_{CTX\text{-}M\text{-}9}$ and $bla_{OXA\text{-}1}$ group genes in clinical *Klebsiella pneumoniae* and *Escherichia coli* strains. *J. Antibiot. (Tokyo).* **2015**, *68*, 725–733, doi:10.1038/ja.2015.68.

34. Qi, C.; Malczynski, M.; Parker, M.; Scheetz, M.H. Characterization of genetic diversity of carbapenem-resistant *Acinetobacter baumannii* clinical strains collected from 2004 to 2007. *J. Clin. Microbiol.* **2008**, *46*, 1106–1109, doi:10.1128/JCM.01877-07.

35. Xia, Y.; Liang, Z.; Su, X.; Xiong, Y. Characterization of carbapenemase genes in *Enterobacteriaceae* species exhibiting decreased susceptibility to carbapenems in a university hospital in Chongqing, China. *Ann. Lab. Med.* **2012**, *32*, 270–275, doi:10.3343/alm.2012.32.4.270.

36. Yong, D.; Toleman, M.A.; Giske, C.G.; Cho, H.S.; Sundman, K.; Lee, K.; Walsh, T.R. Characterization of a new metallo-β-lactamase gene, $bla_{NDM\text{-}1}$, and a novel erythromycin esterase gene carried on a unique genetic structure in *Klebsiella pneumoniae* sequence type 14 from India. *Antimicrob. Agents Chemother.* **2009**, *53*, 5046–5054, doi:10.1128/AAC.00774-09.

37. Liu, Y.Y.; Wang, Y.; Walsh, T.R.; Yi, L.X.; Zhang, R.; Spencer, J.; Doi, Y.; Tian, G.; Dong, B.; Huang, X.; et al. Emergence of plasmid-mediated colistin resistance mechanism MCR-1 in animals and human beings in China: A microbiological and molecular biological study. *Lancet Infect. Dis.* **2016**, *16*, 161–168, doi:10.1016/S1473-3099(15)00424-7.

38. Versalovic, J.; Koeuth, T.; Lupski, R. Distribution of repetitive DNA sequences in eubacteria and application to finerpriting of bacterial enomes. *Nucleic Acids Res.* **1991**, *19*, 6823–6831, doi:10.1093/nar/19.24.6823.
